# Biology and demographic growth parameters of cowpea aphid ( *Aphis craccivora* ) on faba bean ( *Vicia faba* ) cultivars

**DOI:** 10.1093/jis/14.1.120

**Published:** 2014-09-01

**Authors:** A. Soffan, A. S. Aldawood

**Affiliations:** Plant Protection Department, College of Agriculture and Food Sciences, King Saud University. Riyadh, Kingdom of Saudi Arabia

**Keywords:** resistance, whole plant, detached leaf, intrinsic rate of increase, Gazira2

## Abstract

The performance of cowpea aphid, *Aphis craccivora* Koch. (Hemiptera: Aphididae), on five faba bean, *Vicia faba* L. (Fabales: Fabaceae) cultivars was evaluated. Colony development, biology, and demographic parameters were studied to measure the cowpea aphid performance. Two methods, whole plant and detached leaf, were used in these experiments. After 14 d , the number of apterous adult, nymphs, and total cowpea aphids were significantly lower in cultivar Gazira2 and highest on cultivar Misr1. Assuming that low aphid numbers per plant represented high resistance, the order of resistant cultivars was as follows: Gazira2 > Misr > Giza3 Improved > Goff1 > Misr1. Aphid infestation significantly inhibited plant growth compared with uninfested plants, as indicated by factorial analysis using plant height ( *F* = 41.38, *P* < 0.0001). The detached-leaf biological assay showed that the cultivar Gazira2 was less suitable than Misr1 because it had longer prereproductive, reproductive, and post reproductive periods, longer total longevity, and lower number of progeny. Similarly, demographic parameters also justified the suggested lower suitability of Gazira2 compared with Misr1, indicated by significantly lower net reproduction rate, intrinsic rate of increase, finite rate of increase, but longer generation time and doubling time on Gazira2. It was shown that cowpea aphid performed differently on the whole plant as compared with detached leaves. The detached-leaf biological assay is recommended for future experiments because it is more accurate and efficient and it produces reliable data.

## Introduction

The cowpea aphid, *Aphis craccivora* Koch. (Hemiptera: Aphididae), is one of the most common and well-known insect pests throughout the world ( Minks and Harrewijn 1987 , Blackman and Eastop 2006 , Sadeghi et al. 2009 ). Aphids are important piercing-sucking insects that during feeding cause significant loss of a plant’s phloem sap, which is essential for plant growth ( Dixon 1998 ). Indirectly, cowpea aphid also disturbs the photosynthesis process by the presence of fungus on the leaves that is supported by the aphids’ honeydew secretion ( Klingler et al. 2001 , Smith and Boyko 2007 ). Plant damage increases because of the aphids’ role as vectors for numerous plant viruses ( Aldryhim and Khalil 1993 , Smith and Boyko 2007 ), such as faba bean necrotic yellow virus , broad bean yellow mosaic virus, and bean leaf roll virus ( Weigand and Bishara 1991 ).

In the Kingdom of Saudi Arabia, cowpea aphids were first reported in 1989 ( Aldryhim and Khalil 1996 ). They were reported to be abundant during February feeding on at least 29 host plants, most frequently species of Fabaceae ( Aldryhim and Khalil 1993 , 1996). The apterous or alate adult females of the cowpea aphid are readily identified ( Aldryhim and Khalil 1996 ; Stoetzel et al. 1996 ).

Cowpea aphids as a pest of faba bean, *Vicia faba* L. (Fabales: Fabaceae), are increasingly more important because of their higher occurrence in the field and increased deleterious effects on plants ( Weigand and Bishara 1991 ). A short generation time and high fecundity of the aphid cause enormous reproductive potential during a growing season ( Klingler et al. 2001 ). An infestation rate of 71% in the field was reported in Morocco (Diekmann 1982 *in*Weigand and Bishara 1991 , pp. 68). Infestations of black bean aphids, *Aphis fabae* Scopoli *,* stunted the growth of most faba bean cultivars, such as *V. faba* Major and Aquadul-ce, which led to a decrease in shoot fresh, dry weight, leaf area, and plant height. Infestation during the early stages of plant growth may kill the plant ( Shannag and Ja'far 2007 ).

Many attempts have been made to control cowpea aphid, mostly by insecticides; however, the increasing awareness of environmental and human health hazards has led researchers to develop alternative control measures with integrated pest management (IPM) ( Shannag and Ja'far 2007 ). Joplin (1974) and Schoonhoven et al. (1998) estimated that the use of resistant plants has increased yields by 120 fold. Although partial resistance is usually possible, it is difficult to achieve a complete resistance to a particular insect species. From the IPM point of view, this is an advantage because it poses weaker selection pressure on the insect population to overcome host plant resistance. Therefore, the use of plant resistance should be combined with other IPM tactics ( Schoonhoven et al. 1998 ).

Resistant faba bean cultivars against aphids have been developed, such as *V. faba* Minor and *V. faba* landrace V51, which are tolerant and resistant to *A. fabae* and *A. craccivora* , respectively ( Laamari et al. 2008 , Shannag and Ja'far 2007 ). Because aphids can overcome resistance factors of selected cultivars, additional studies of resistant mechanisms of other faba bean cultivars are urgently needed.

In this experiment, we investigated possible resistance traits in some selected faba bean cultivars against cowpea aphid. We examined colony development and biological‒demographic parameters with two methods, whole plant and detached leaf.

## Materials and Methods

This study was conducted at the Economic Entomology Research Unit Laboratory, Plant Protection Department, College of Food and Agriculture Sciences, King Saud University, Riyadh, Kingdom of Saudi Arabia. All experiments were conducted in a growth chamber maintained at 26 ± 0.1°C, 44 ± 0.1 % RH (means ± SE) with a photoperiod of 16:8 L:D h (recorded by HOBO data loggers; ONSET Co., Bourne, MA).

### Plant material

Five faba bean cultivars (Misr1, Misr, Giza3 Improved, Goff1, and Gazira2) were used for the experiments. Faba bean seeds were obtained from the Legume Research Unit (LRU) Plant Production Department, College of Food and Agriculture Science, King Saud University. Seeds were soaked in water for 48 h and then germinated in a mixture of sand and peat moss (1:1) growth medium. After 1 wk, seedlings were transplanted to plastic pots (11 cm diam14 cm tall). Four granules per pot of complete fertilizer (nitrogen: 12%, phosphorus: 12%, potassium 17%; BASF-Asoco Agro, Limburgerhof, Germany) was applied in the growth medium once at seedling stage (19 d). The pots were drenched with 150 mL water once every 2 d.

### Cowpea aphids

Experimental cowpea aphids were obtained from a colony that was collected from alfalfa plants grown in Al Amaria, Riyadh (46°31'5.5518"N and 24° 48'40.179"E). Cowpea aphid voucher specimen was deposited in King Saud Museum of Arthropods (KSMA), King Saud University. A single mother of apterous adult aphid was used to initiate cowpea aphid culture on the faba bean cv. Misr. Prior to the experiment, the cowpea aphid culture had been running for 8 mo. New faba bean seedlings were provided continuously to replace old ones for the maintenance and continuous growth of aphid cultures.

### Aphid colony-development test

Two-wk-old, 6-8 cm plants were used for experimentation. Plants were acclimatized in 600 mL plastic pots. Apterous nymphs of *A. craccivora* of the same age were obtained from the aphid culture synchronization by rearing them on detached leaves of faba bean cultivars Misr. Ten 3-5-d-old nymphs were introduced on each plant and covered with clear plastic PVC tubes (30 cm high by 10 cm diam) and ventilated using muslin cloth (15 x5 cm). The plastic tube allowed aphids to move freely to sections of the plant for feeding, while preventing escape. Uninfested plants were provided to all of the above but without aphid infestation. Ten plants were used for infested and uninfested plants. The experiments were terminated 14 d after infestation by cutting the plant from the base. Plant height and aphid numbers consisting of adults (alatae and apterae) and nymphs (alatae and apterae) were counted as parameters. Aphids were collected from the plants and preserved in 75% ethanol until the counting was ready.

### Aphid biological study

Whole plants and detached leaves were distinguished in the biological study for all cultivars. The whole-plant study was conducted by using a modified clip cage (6x3 cm) to cover the selected leaf completely; the aphids were observed and allowed to move freely in entire leaf area.

A 4 *x 2* cm muslin cloth mesh on the clip cage provided air circulation. Bamboo or metal sticks supported the clip cage on the plant. In the detached-leaf study, aphids were provided with a cut leaf. The leaf was kept fresh by capping the leaf base with moist cotton. A clear plastic elliptical container (215 mL) with 20 cm ^2^ muslin cloth for aeration was used in the detached-leaf study.

Whole-plant and detached-leaf studies were initiated by introducing a single apterous adult mother onto a single leaf of the three-leaf stage of faba bean plant. After 4‒6 h, all aphids were removed with a camel hair brush (0 size), except for one newly born aphid. Nymphal developmental periods (prereproductive period, reproductive period, post-reproductive period, and total longevity periods) were measured daily, and the total number of progeny counted.

### Experimental design and data analysis

All experiments were arranged in a complete randomized design (CRD). Ten replicates of each faba bean cultivar were used. All the data were analyzed using SAS program ver. 9.2 (2008, SAS Institute, Cary, NC). Normality distribution was tested using PROC UNIVARIATE with Shapiro-Wilk method before ANOVA analysis. Parametric one-way ANOVA test was performed for colony development study ( Tables 1 and 2 .) with PROC GLM, followed by multiple comparison using Least Significant Difference (LSD) test (α: 0.05). In the biological study and demographic study ( Tables 3 and 4 ), nonparametric oneway ANOVA Kruskal-wallis was performed using PROC NPAR1WAY, because the study did not meet normal distribution conditions. Demographic parameters were calculated following Birch (1948) . Proportion of individuals alive at age x (lx) and number of females progeny produced per female during age interval x (mx) were determined from daily data observation. The following demographic parameter then was calculated: (1) intrinsic rate of increase (rm) rm = (lnRo)/T; (2) net reproduction rate (Ro), Ro = Σ lxmx; (3) mean generation time (Tc), Tc = Σ(lxmx)x/Σ(lxmx); (4) finite rate of increase, λ = exp(rm); and (5) doubling time, Td = ln(2)/rm. The jackknife technique was used to estimate mean demographic parameters of lxmx of the life table and the standard errors (SE). Jackknife analysis removes one observation at a time from the original data set and recalculates the statistic of interest from the truncated data set. This method can estimate Ro, Tc, rm, λ, and Td, with their respective jackknife variances and confidence intervals ( Myers et al. 2005 , Chen et al. 2010 ).

## Results

The numbers of aphids after 14 d of the colony development test varied among five faba bean cultivars ( Table 1 ). Assuming that lower aphid numbers reflect higher level of resistance, the order of resistance levels were Gazira2 > Misr > Giza3 Improved > Goff1 > Misr1. The cultivar Gazira2 had the lowest total numbers of aphids, and Misr1 had the highest. Nymph and adult apterous cowpea aphid numbers on Gazira2 were significantly lower than on Misr1; however, adult alatae numbers on Gazira2 and Misr1 were not significantly different ( Table 1 ).

**Table 1.**
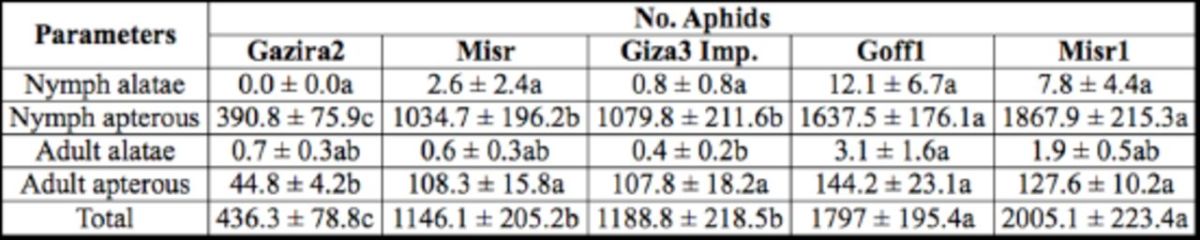
Cowpea aphid, *Aphis craccivora,* colony-development test for 14 d on five *Vicia faba* culti-vars (means ± SE). ^1,2^

Means followed by the same letter in the same row are not significantly different at LSD, a= 0.05. ^2^ Parametric one-way ANOVA was performed for the above analysis.

Plant response to aphid infestation was evaluated by measuring the plant height ( Table 2 ). Misr1 exhibited the highest susceptibility to aphid infestation among cultivars, indicated by the lowest height of the infested plants, and had significant difference compared with un-infested plants ( *P* < 0.01). There were no significant differences between plant heights of infested and uninfested cultivars Gazira2 and Goff1. It is interesting to note that the cultivar Goff1 supported relatively high numbers of aphid ( Table 1 ), but the plant height was not significantly different, which indicates the possible occurrence of tolerance ( Table 2 ).

**Table 2.**
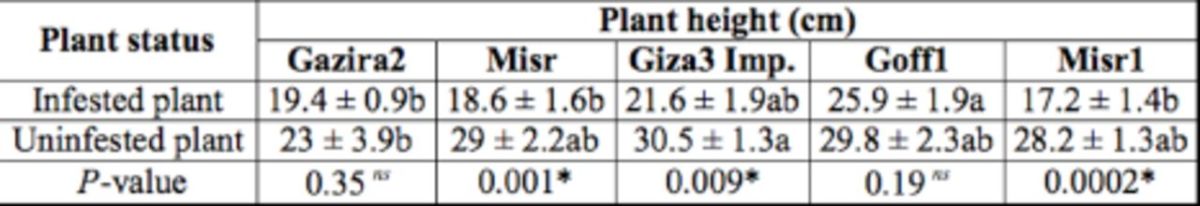
Plant height (cm) from the *Aphis craccivora* colony-development test on five *Vicia faba* cultivars (Means ± SE).1,2,3

Means followed by the same letter in the same row are not significantly different at LSD, α = 0.05

^2^ Means in the same column for each cultivar comparing infested and uninfested plant accompanied with *P* -value, asterisk (*) for significant difference, *ns* is nonsignificant at LSD, α = 0.05.

^3^ Parametric one-way ANOVA was performed for the above analysis.

Factorial analysis indicated that uninfested plant height as main effect had significantly higher value compared with infested plants ( *F* = 41.38, *P* < 0.01). The effect of cultivar, regardless of the infestation status, showed sig-significant difference ( *F* = 3.28, *P* < 0.01). Nevertheless, the interaction between the two factors (cultivars and infestation factors) was not significantly different ( *F* = 1.22, *P* = 0.3).

Aphids had longer total longevity and fewer progeny in the whole-plant biological assay ( Table 3 ) on the cultivar Gazira2 as compared with Misr1, although there was no significant difference. In the detached-leaf biological assay, the cultivar Gazira2 had a longer prereproductive, reproductive, post-reproductive, and total longevity period as compared with Misr1, whereas the number of progeny was lower as compared with Misr1. Generally, cowpea aphid performance on whole plants was different as compared with the detached-leaf assay.

**Table 3:**
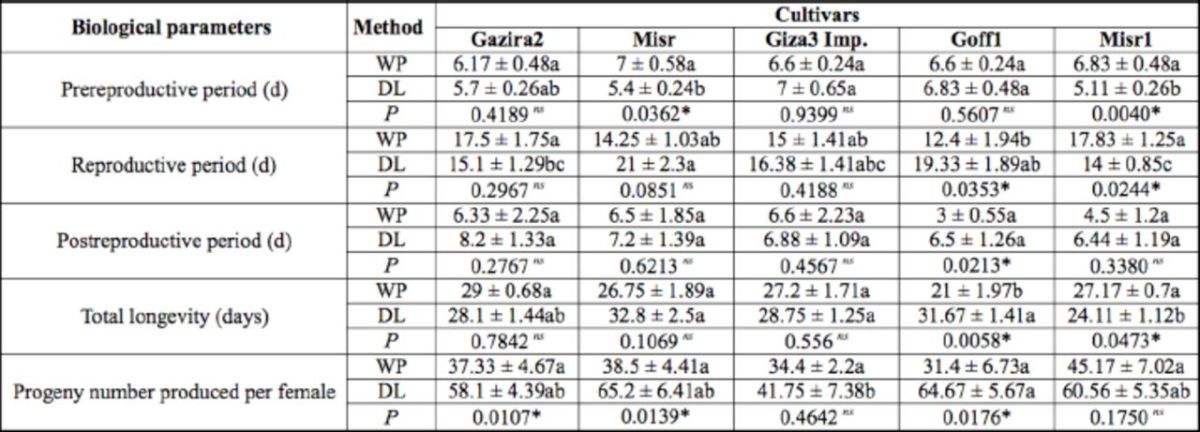
Biological study of apterous viviparous female of cowpea aphid, *Aphis craccivora,* on whole plants and detached leaves of five *Vicia faba* cultivars (means ± SE). ^1^^23^

^1^ WP:Whole plant; DL: Detached leaves; *P: P* -value.

^2^ Means followed by the same letter in the same row are not significantly different at LSD, a = 0.05.

^3^ Means in the same column for each parameter comparing whole plant (WP) and detached leaf (DL) accompanied with *P* -value,

asterisk (*) for significant difference, *ns* is nonsignificant at LSD, a = 0.05.

^4^ Nonparametric one-way ANOVA Kruskal Wallis was performed for the above analysis

**Figure 1.**
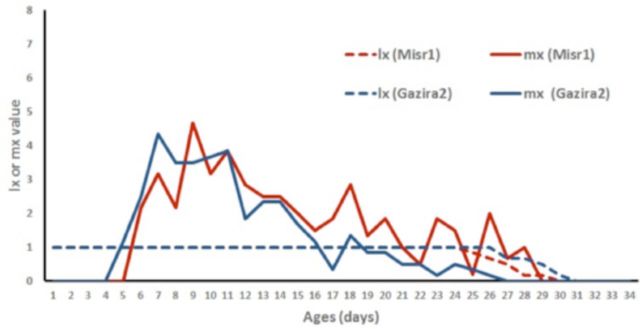
Survival (lx) and reproduction (mx) rate of cowpea aphid, *Aphis craccivora,* on whole plants of two cultivars of *Vicia faba,* Gazira2 and Misr1.

**Figure 2.**
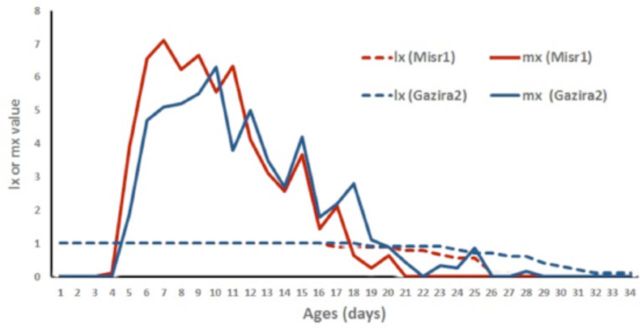
Survival (lx) and reproduction (mx) rate of cowpea aphid, *Aphis craccivora,* on detached leaves of two cultivars of *Vicia faba,* Gazira2 and Misr1.

Demographic parameters study ( Table 4 ) on whole-plant biological assay showed that cultivar Gazira2 had a significantly lower net reproduction rate (Ro) compared with Misr1; other parameters had no clear relationship with the lesser suitability character of Gazira2 compared with Misr1. On detached leaf, all the demographic parameters supported that Gazira2 was less suitable for the aphid compared with Misr1, indicated by the significantly lower net reproduction rate (Ro), intrinsic rate of increase (rm), and finite rate of increase (X), and longer generation time (T) and doubling time (Td). Similar to biological parameters ( Table 3 ), cowpea aphid demographic performance on whole plant and detached leaf was different ( Table 4 .). Figures 1 and 2 show that Misr1, either on detached leaf or whole plant, seems preferable to the aphid compared with Gazira2, indicated by higher fecundity rate (mx) on Misr1.

**Table 4:**
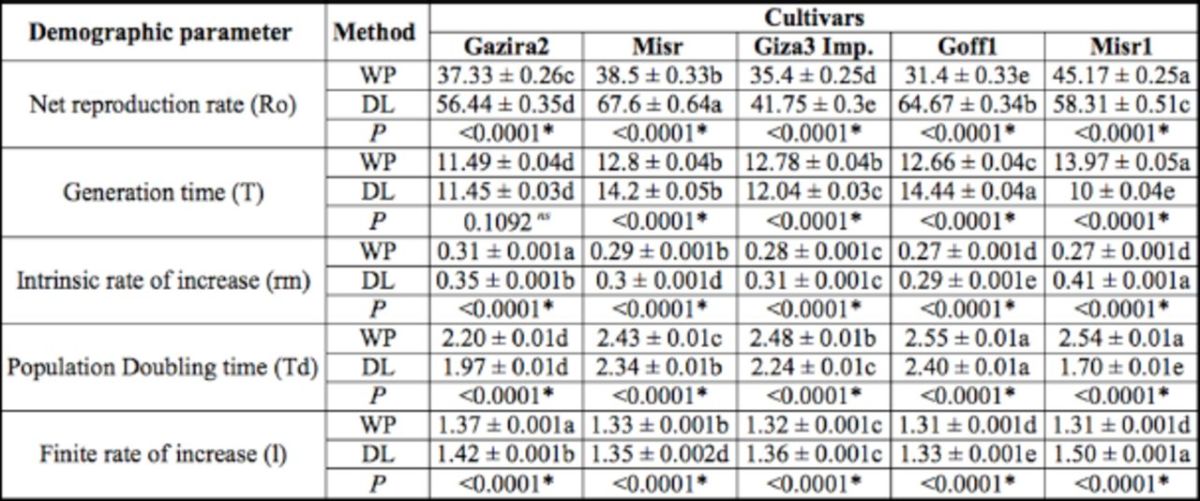
Demographic parameters for cowpea aphid, *Aphis cracciv ora,* on five *Vicia faba* cultivars (means ± SE). ^1^^23^ , ^4^

## Discussion

The colony development study was conducted initially to obtain general information about the potential resistant characters that might be present among five faba bean cultivars. Studies showed that the tested cultivars differed in their suitability for the cowpea aphid development. All the parameters in colony development assay indicated that the order of cultivar resistance from the highest to the lowest was Gazira2 > Misr > Giza3 Improved > Goff1 > Misr1. Results obtained from the colony development assays probably reflect a similar phenomenon in the field because aphids were allowed to move freely and to feed in any part of the plant ( Alvarez et al. 2006 ). Factors such as plant height, leaf surface, leaf size, or leaf color supposedly affect the results of colony development assays ( Bernays and Chapman 1994 ). Some differences in host plant quality among cultivars due to genetic variation or environmental factors also can determine cowpea aphid performance ( Islam and Shunxiang 2007 ). It is important to note that the Gazira2 has the shortest plant height among the cultivars, which might have correlated with the aphids preferring it the least ( Table 2 .). It also was shown that adult alatae numbers in Gazira2 were not significantly different as compared with Misr1, which could indicate the unsuitability of Gazira2 as host plant. Cowpea aphid infestation significantly reduced plant height *(F =* 41.38, *P* <0.01). Significant difference in height between infested and uninfested plants occurred in most cultivars, except Gazira2 and Goff1. The highest total number of aphids in Misr1 was positively correlated with the most significant difference between the height of the infested and uninfested plants.

Whole-plant biological parameters ( Table 3 .) indicated that aphids had longer total longevity and fewer progeny on cultivar Gazira2 compared with Misr1, although it did not differ significantly. This may reflect that the cultivar Gazira2 exhibited higher resistance compared with Misr1. However, other parameters did not give a clear relationship to support this conclusion. Longer total longevity and fewer progeny are essential parameters indicating suitability of a host plant, especially for sucking insects ( Awmack and Leather 2002 , Bernardi et al. 2012 ).

Most of the biological parameters evaluated in the detached-leaf assay ( Table 3 ) showed that the cultivar Gazira2 was less suitable for cowpea aphid development compared with Misr1, indicated by longer prereproductive, reproductive, post-reproductive, and total longevity periods, and fewer aphid progeny. Bernardi et al. (2012) reached a similar conclusion on the resistant strawberry cv. Aromas infested by green aphid, *Chaetosiphon fragaefolii* (Cockerell). Using the detached-leaf method for biological assay is recommended by some researchers because of its efficiency ( Sharma et al. 2005 , Smith 2005 , Michel et al. 2010 ). We noted that the detached-leaf assay was easier to handle and provided more accurate information because all of the environmental parameters were controlled.

When comparing whole-plant and detached-leaf methods in the biological study, results varied among cultivars for all parameters, but the progeny number parameter was significantly higher in the detached-leaf assay. This result gave a precaution in using detached leafs in resistant screening assays for faba beans, probably because of the difference in tissue properties. However, these studies might be useful for determining the persistency of resistant characters, as shown in Michel et al.’s (2010) soybean cultivar study against soybean aphid, *Aphis glycines* (Matsumura), which concluded that the resistant character was retained in detached leaves in PI 243540’ but it was lost in PI 567301B.

Net reproduction rate of whole plant faba bean cultivar Misr1 was significantly higher than Gazira2 ( Table 4 ). If the net reproduction rate value is used as the only parameter, it can be concluded that whole plant Gazira2 was less suitable for the aphids than Misr1. Net reproduction rate value is important in representing the host plant quality and the capacity of a female producing the progeny ( Bernardi et al. 2012 ). It was important to note that cowpea aphid fecundity in whole-plant faba bean cv. Misr1 remained high at the end of their reproduction period ( Fig. 1 ). This fact deviated from the general assumption that the progeny are supposed to peak at the beginning of the reproduction period ( Wyatt and White 1977 ). These phenomena result in the higher value of generation time and decrease the intrinsic rate of increase value of Misr1 compared with Gazira2.

The detached-leaf demographic parameters study gave reliable results and supported the suggested less suitability of cultivar Gazira2 compared with Misr1, indicated by significantly lower net reproduction rate, intrinsic rate of increase, and finite rate of increase, but longer generation time and doubling time, compared with Misr1. Those demographic results are important to justify resistant characters of certain cultivars, such as in strawberry cv. Aroma against green aphid ( Bernardi et al. 2012 ). In addition, demographic parameters can be used to measure the suitability of a host plant for certain insects, such as in some tomato cultivars against *Bemisia tabaci* (Gennadius) ( Islam and Shunxiang 2007 ), the suitability of chickpea pods for *Helicoverpa armigera* (Hubner) ( Dabhi and Patel 2007 ), the suitability of different host plants for glassy-winged sharpshooter, *Homalodisca vitripennis* (Germar) ( Chen et al. 2010 ), and for evaluating artificial diet efficiency ( Wittmeyer and Coudron 2001 ). Cowpea aphid performance measured by demographic parameters ( Table 4 ) has similar conclusions as the biology study ( Table 3 ), indicating that either study can be used to generate the conclusion. Overall, those results suggested that the use of faba bean cultivar Gazira2 should decrease the cowpea aphid population.

### Conclusion

The colony development study ranked the resistance level among cultivars as Gazira2 > Misr > Giza 3 improved > Goff1 > Misr1. Demographic parameters in the detached-leaf study showed that Gazira2 is less preferred than Misr1 because of lower net reproduction rate, intrinsic rate of increase, and finite rate of increase, but longer generation time and doubling time. The detached-leaf biological assay is preferable because it is more reliable and produced similar results as the colony development study.
